# Social Crowding during Development Causes Changes in GnRH1 DNA Methylation

**DOI:** 10.1371/journal.pone.0142043

**Published:** 2015-10-30

**Authors:** Sebastian G. Alvarado, Kapa Lenkov, Blake Williams, Russell D. Fernald

**Affiliations:** Biology Department and Neuroscience Institute, Gilbert Hall, Stanford University, 371 Serra Mall, Stanford, Palo Alto, Califorinia 94305, United States of America; Università di Napoli Federico II, ITALY

## Abstract

Gestational and developmental cues have important consequences for long-term health, behavior and adaptation to the environment. In addition, social stressors cause plastic molecular changes in the brain that underlie unique behavioral phenotypes that also modulate fitness. In the adult African cichlid, *Astatotilapia burtoni*, growth and social status of males are both directly regulated by social interactions in a dynamic social environment, which causes a suite of plastic changes in circuits, cells and gene transcription in the brain. We hypothesized that a possible mechanism underlying some molecular changes might be DNA methylation, a reversible modification made to cytosine nucleotides that is known to regulate gene function. Here we asked whether changes in DNA methylation of the *GnRH1* gene, the central regulator of the reproductive axis, were altered during development of *A*. *burtoni*. We measured changes in methylation state of the *GnRH1* gene during normal development and following the gestational and developmental stress of social crowding. We found differential DNA methylation within developing juveniles between 14-, 28- and 42-day-old. Following gestational crowding of mouth brooding mothers, we saw differential methylation and transcription of GnRH1 in their offspring. Taken together, our data provides evidence for social control of GnRH1 developmental responses to gestational cues through DNA methylation.

## Introduction

To survive, it is essential for animals to respond quickly to changes in their environment. Over longer time-scales, environmental changes can produce life-long effects on both behavior and physiology. In humans, for example, variations in quality of early life experiences have been associated with a number of psychological consequences such as increased depression and anxiety [1, 2]. Such changes in behavior are often accompanied with a suite of anatomical [3] and molecular marks [4, 5]. One mechanism known to regulate transcriptional gene function is epigenetic modification. Epigenetic modifications such as histone modification and DNA methylation [6, 7] are known to have important roles during cell division, imprinting, and differentiation [8, 9]. These epigenetic changes, specifically DNA methylation, can also occur in response to environmental events, both during early development and in later life [10–13]. In rodents, for example, variations in the quality of early maternal care has been correlated with stress reactivity and is thought to be regulated, at least in part, by changes in the methylation state of the glucocorticoid receptor (*GR*) gene [14]. In humans, prenatal exposure to maternal depression during pregancy has been associated with differential methylation of the human GR, *NR3C1*, [15] and suicide completers with a history early-life abuse were found to have increased methylation of rRNA and *NR3C1* [16, 17]. Furthermore, unique signatures of DNA methylation accompany and mediate transgenerational effects underlying behaviors related to stress [18, 19] underlining their importance in generational adaptation to stress.

Here, we asked whether there was a DNA methylation response to social stress, particularly related to reproductive activity mediated by the brain-pituitary-gonadal (BPG) axis. The brain regulates sexual maturation and reproduction [20] via this axis by delivering gonadotropin-releasing hormone 1 (*GnRH1*) from the brain to the pituitary. GnRH1 is a decapeptide highly conserved in all vertebrates [21, 22], that is synthesized in neurons located in the basal forebrain or pre-optic area (POA) [23, 24] that signals the pituitary to release gonadotropin hormones LH and FSH into the bloodstream. The reproductive pathway is highly responsive to stress with a consequent strong inhibitory effect [25, 26].

Here, we studied gene-specific DNA methylation and its effect on transcription in response to social stress in females of an east African cichlid fish species, *Astatotilapia burtoni*. In this species, social interactions regulate the male reproductive system directly. There are two distinct, socially reversible, phenotypes: dominant males that are reproductively competent and subordinate males that are reproductively incompetent. The difference in social status is evident with the increase in *GnRH1* expression that occurs when animals ascend in social status, become brightly colored, change their behavioral repertoire, and become reproductively capable [27, 28]. In females, there is an equivalent change in GnRH1 regulation but it follows an internal cycle with peak *GnRH1* expression just prior to spawning [3]. A female becomes gravid approximately on a 6-week cycle and once it mates with a male, collects the eggs in her mouth where she broods them for two weeks before releasing them as fry (Fernald, 1977).

We measured the methylation levels of *GnRH1* during normal development as well as under conditions where the mother was stressed through crowding. We examined the CG nucleotide sites where methylation occurs by sampling developing fry at different times and subsequently measured resulting *GnRH1* DNA methylation patterns across the whole animal. To see whether *GnRH1* methylation could be affected by an adverse early-life environment, we housed brooding females either at low density or in crowded aquaria. *GnRH1* methylation and transcription levels were then measured in the progeny of females crowded while brooding and compared with methylation and transcription levels in normal breeding females at 2 weeks post-fertilization.

## Materials and Methods

### Animals

All animal work was performed in compliance with the animal care and use guidelines of the Stanford University Administrative Panel for Laboratory Animal Care (Protocol 9882).

Subjects were laboratory-bred cichlid fish *A*. *burtoni*, derived from stock wild-caught in Lake Tanganyika, Africa [29]. Animals were housed in aquaria under conditions that closely mimic their natural habitat (28°C, pH 8.0, 12 hours light and 12 hours dark cycle with full spectrum illumination, and constant aeration), and fed daily with cichlid pellets and flakes approximately one pellet and one flake per adult fish (AquaDine, Healdsburg, CA). Juvenile animals were fed ground flakes with a diameter smaller than the width of their mouths and their diet was supplemented with brine shrimp. Aquaria contained gravel substrate and semi-cylindrical terra-cotta pots that served as shelters and spawning territories.

### Raising juvenile animals

Eight breeding females were raised in large aquaria (151 L) or small aquaria (30 L) for non-crowded and crowded social paradigms, respectively. Non-crowded breeding tanks contained 2 size-matched males between 3 cm and 5 cm with 2 spawning shelters. Crowded tanks, housed 8 females and, and two males male between 3–5 cm with one spawning shelter.

**Fig 1:**
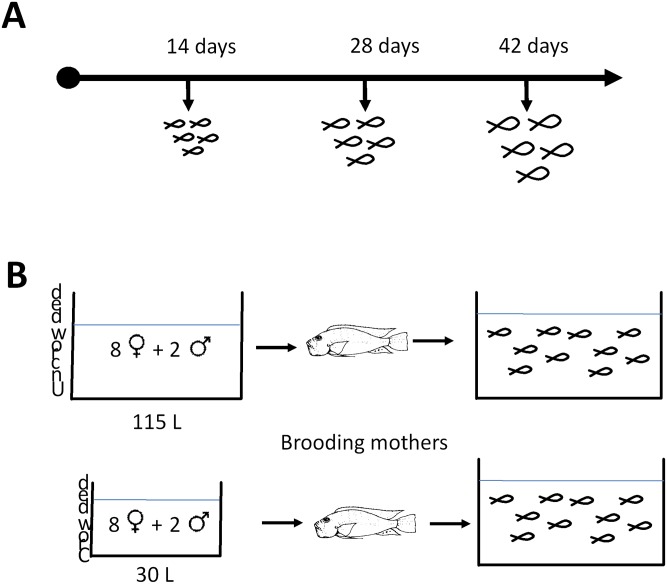
Schematic of experimental designs used to characterize development **(A)** for 14-, 28- and 42- day old fry (n = 25) and **(B) comparison of uncrowded and crowded conditions**.

After spawning, *A*. burtoni females brood their young in their mouths before releasing fry at ~14 days post-fertilization. Breeding tanks were monitored several times a day and mouth- brooding females were identified by daily visual inspection. At two weeks post-fertilization, each mouth brooding female was transferred into a smaller aquarium (30L). While the number of fry in a single mother can vary between 15–30, the total number of fry from each mother for analyses post-day 14 were capped at n = 25 per tank. Upon transfer, adult females expelled their brood and were removed from the tank. We used a crowding paradigm early in development and assayed changes at 14 days. Animals for the 14-day time point were immediately collected and 25 fry were left in each tank (Fig 1A). This was done early in development before adult-specific behaviors could affect the regulation of GnRH1. For crowded fry, 14-day-old animals were immediately collected and 25 fry were transferred to small 1L aquaria. The volume for these “crowded” growing tanks was determined by photographing and measuring the volume that a brood of this size and age typically occupies and then dividing it in half (Fig 1B).

### Tissue Preparation

Upon collection, fry were placed in iced water and standard length (+/- 1mm) and weight (+/- 0.01 g) were measured. Animals were either sacrificed by rapid cervical transection or flash frozen whole in a methanol/dry ice bath. DNA and RNA (DNeasy/RNeasy Blood and Tissue kit, Qiagen, Valencia, CA) was extracted and assayed from entire heads and bodies, separately. Measurements of DNA methylation between heads and bodies separately showed no significant differences (S1 Fig), allowing our comparison between the heads of 28–42 day-old juveniles and whole body of 14 day-old juveniles throughout our study. Separated heads/bodies or entire specimens were place in 1.5mL tubes and stored at -80°C until processing.

### DNA methylation profiling and expression analysis

**Fig 2:**
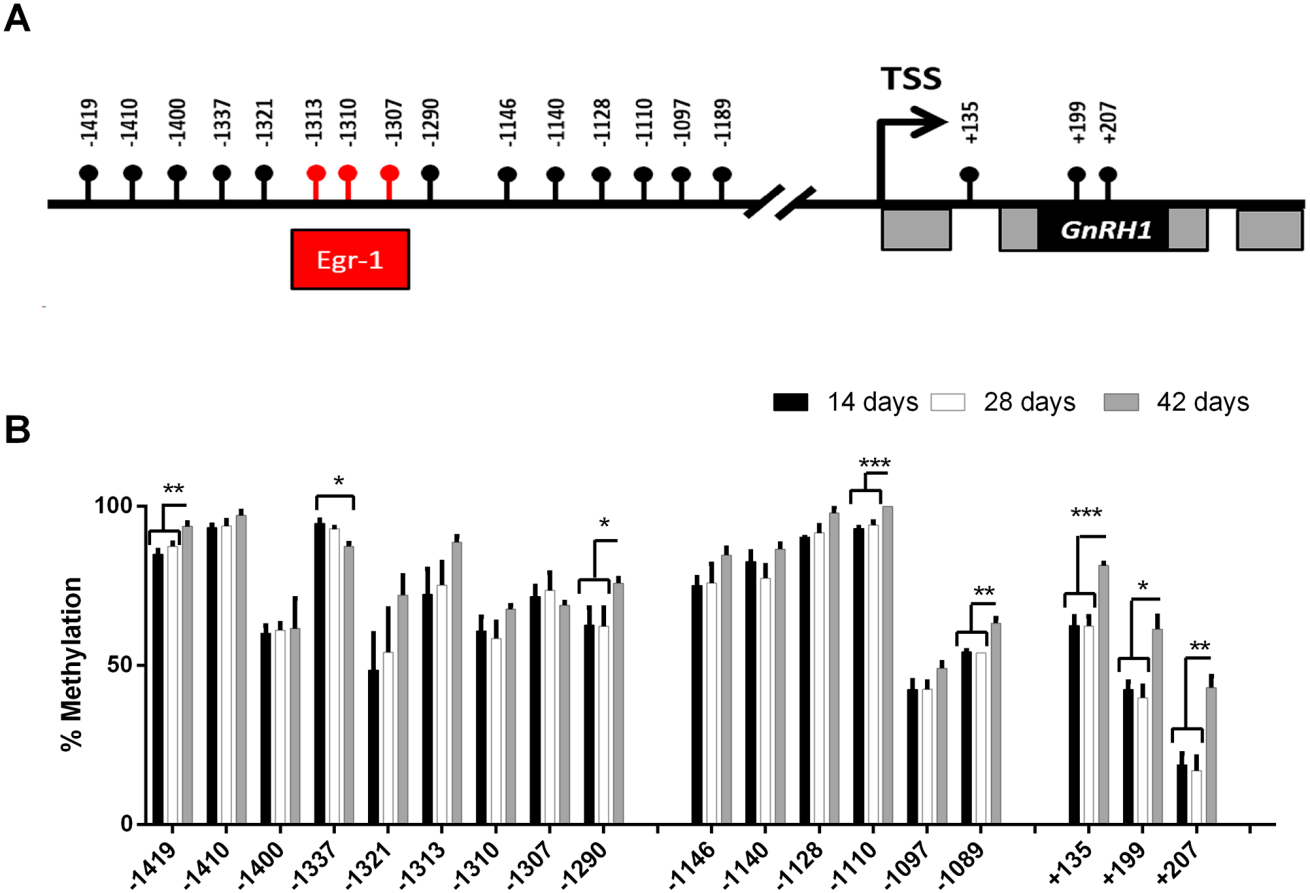
DNA methylation of the *GnRH1* gene during development **(A)** Physical map of the GnRH1 promoter and coding region. Location of the *Egr-1* binding site in red. Coding regions in black, non-coding in gray. TSS is the transcription start site **(B)** Fractional methylation in the GnRH gene at 18 positions on the GnRH gene for three different developmental time points, 14- (white), 28- (grey), and 42-days (black) * = p<0.05, ** = p<0.001, *** = p<0.0001. (n = 5–7 /group). Error bars represent s.e.m.

Purified DNA was bisulfite converted as previously described by Clark et al [30]. Primers were directed against CG rich regions in the promoter and coding region of the *A*. *burtoni GnRH 1* gene (Genebank accession number AF076961; See Fig 2). Primers were designed to exclude any CpG dinucleotides so that methylated and unmethylated sequences were amplified with the same efficiency (Table 1). The sequencing template was prepared using the standard protocol (Stanford Protein and Nucleic acid core facility [31]). Briefly, for each PCR amplification, one inner primer was biotinylated at 5’ end at allow for immobilization of the amplicon. 15–20 μl of each biotinylated PCR product was combined with and immobilized on streptavidin coated sepharose beads (Dynabeads M280, Dynal, Oslo, Norway). The immobilized DNA was treated with sequential washes of 70% ethanol, 0.2 M NaOH (for denaturation of the DNA), and Tris buffer. The single-stranded DNA target was then hybridized to specific sequencing primers. Pyrosequencing was performed using the PyroMark Q96 DNA sequencing system (Qiagen, Valencia, CA) according to manufacturer’s instructions. For analysis of methylation at individual CpG sites, data are presented as percent methylation averaged across samples. cDNA libraries were built from 2μg of extracted RNA (quantified by Nanodrop spectrometer) using the iScript system according to manufacturer’s instructions (Biorad, Pleasanton, CA). qPCR experiments were then performed in triplicate using Ssofast Supermix (Biorad, Pleasanton, CA) according to the manufacturer’s guidelines with primers designed across exon boundaries. qPCR was performed using primers specific for *GnRH1* and were standardized to the geometric mean of *rpl32* and *gapdh* (Table 1). All differences between groups were analyzed using an unpaired Student’s t-test.

**Table 1:** Primer sequences and melting temperatures for QPCR.

Primer	Sequence (5’-3’)	Tm
**qPCR**		
*rpl32* forward	CGGTTATGGGAGCAACAAGAAAAC	60°C
*rpl32* reverse	GGACACATTGTGAGCAATCTCAGC	
*gnrh1* forward	CAGACACACTGGGCAATATG	60°C
*gnrh1* reverse	GCCCACACTCGCAAGA	
*gapdh* forward	AAACACACTGCTGCTGCCTACATA	60°C
*gapdh* reverse	CACACAAGCCCAACCCATAGTC	
**Pyrosequencing**		
GnRH1 for (-1419 to -1290)	AGATTGAAAGTTGGTAATTGAGAA	48°C
GnRH1 rev (-1419 to -1290)	TTATCTTTTTCCAAATACACCATC	
Seq Forward	ATTGAGAAGATTTTATGTAG	
Seq Forward	AAAGAAAGTGGTATTAATAG	
GnRH1 for (-1146 to -1189)	AGTTTAAGGTTTTTTAAATGGG	48°C
GnRH1 rev (-1146 to -1189)	ACAACAAACATAACCAAACCATTA	
Seq Forward	AAGGTTTTTTAAATGGGT	
GnRH1 for (+135 to +246)	TTGGAGTTAAATGGGGGAAAAA	48°C
GnRH1 rev (+135 to +246)	TCCAAATCCCTCTTCCCTCCTA	
Seq Reverse	TCTTCCCTCCTAAACTCA	
Seq Reverse	TTCTAAAAATAAAACTTCAC	

## Results

### GnRH 1 promoter and coding methylation during development

We measured GnRH1 methylation in *A*. *burtoni* fry at 14, 28, and 42 days post-fertilization (Fig 2A). We chose 15 CG sites within a ~1.5 Kb window upstream of the *GnRH1* transcriptional start site (TSS) and 4 CG sites ~300bp downstream of the TSS (Fig 2A). With the exception of one CG site (-1337), significant *GnRH1* hypermethylation was observed during development at eight CG sites (-1419, -1290, -1110, -1189, +135, +199, and +207) between both 14-day and 28-day old juveniles with 42-day old juveniles (Fig 2B). This hypermethylation event was the largest within the coding region of GnRH1 ranging between 15–30% differences in methylation (Fig 2B).

### Effect of maternal social crowding on progeny

**Fig 3:**
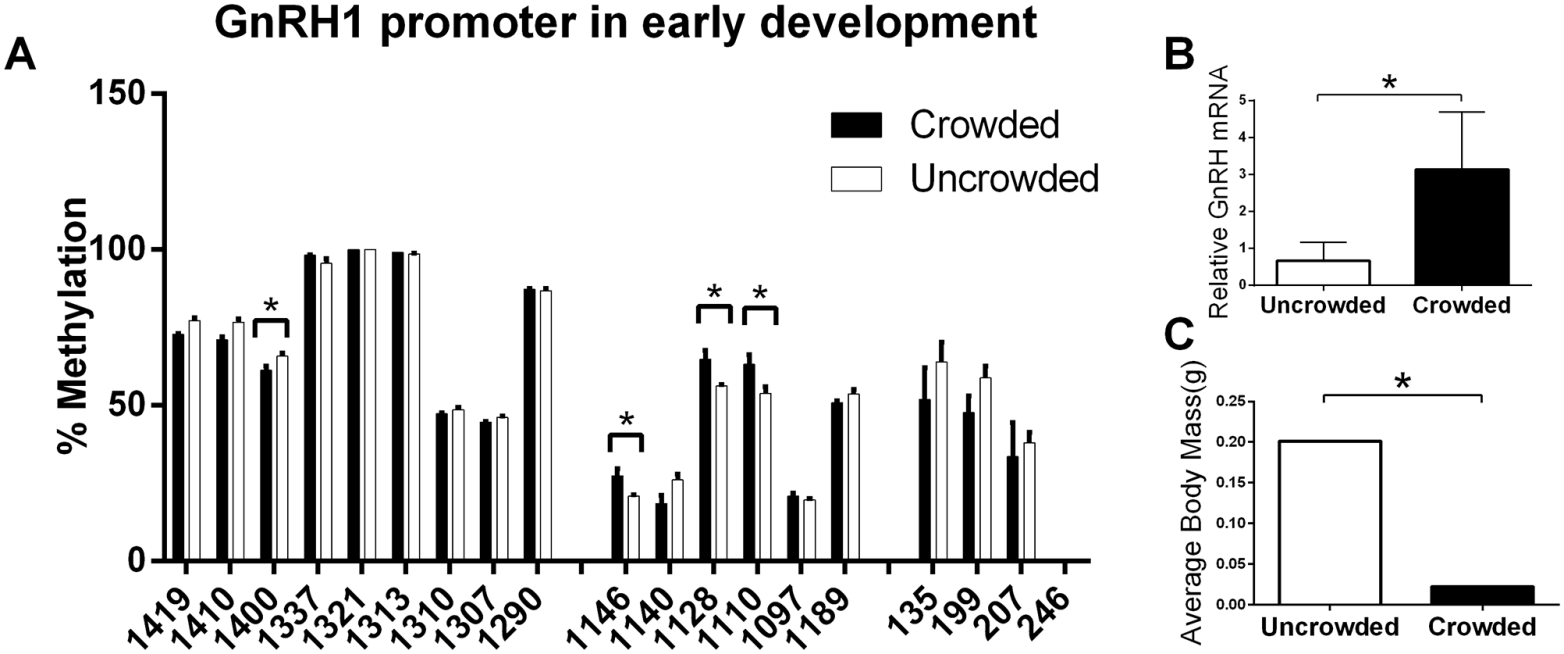
Social crowding results in hypomethylation and silencing of the *GnRH1* gene and decreased body mass **(A)** Bisulfite map of methylation in the *GnRH1* gene and 1.5kb upstream of its transcriptional start site (TSS) from uncrowded (white) and crowded mothers (black) **(B)** Relative expression of *GnRH1* from 14-day-old fry following gestational crowding. **(C)** Body mass seen in 42-day-old fry following gestational and developmental crowding. * = p<0.05. (n = 5–7 /group). Error bars represent s.e.m. Unpaired student’s T-test.

We found that fry from crowded mothers had a hypomethylated GnRH1 promoter versus those from uncrowded mothers at the 14-day-old time point. Specifically, CpGs -1146, -1128, and -1110 were shown to decrease in methylation within maternally and developmentally crowded individuals (Fig 3A). Furthermore, this hypomethylation event was accompanied with a marked increase of GnRH1 transcription (Fig 3B). Between 14- and 28-day-old fry, we were not able to accurately determine a significant difference in size between each group, however, 42-day-old fry from crowded mothers raised in crowded tanks had on average ~18% the body mass of fry from non-crowded mothers and raised in non-crowded tanks (Fig 3C).

## Discussion

To understand possible mechanisms through which the early life environment can exert long-term effects on an organism, we analyzed DNA methylation of the promoter for the reproductively essential *GnRH1*, a key regulator of reproductive development in all vertebrates. Our study has three main findings. Within whole heads and juveniles we found that methylation in the *GnRH 1* promoter and coding region of *A*. *burtoni* remained constant between 2 and 4 weeks post-fertilization but had increased significantly at 6 weeks during development. Second, we found that at 2-weeks post fertilization, immediately after the end of mouth brooding, progeny of gestationally crowded females had significantly decreased methylation levels in the coding region of *GnRH1* and decreased transcription of mRNA. Third, we demonstrated that a sustained social crowding in the maternally crowded progeny resulted in the physiological outcomes of long-term low body mass.

While many genes are known to be affected by social cues we limited our investigation to *GnRH1* because it is regulated by social status in adults and has been thoroughly characterized in *A*. *burtoni*. *GnRH1* governs reproductive development via the hypothalamic-pituitary-gonadal axis by regulating the production of gonadotropins, which in turn controls the production of gonadal steroid hormones. *GnRH1* expression is regulated by a large number of transcription factors [32], including the glucocorticoid receptor GR [33] and the immediate early gene *egr-1* [34], which both have putative binding sites in the promoter region of *A*. *burtoni* [27]. Post-transcriptional regulation also plays a significant role in the control of *GnRH1* mRNA levels [35], allowing for a rapid response to social opportunity [5]. GnRH peptide is released from specific GnRH neurons in a pulsatile manner, synchronized with the release from neighboring neurons [36–38].

We interpret our results to mean that crowding and its effect on development [39] may be regulated through the reproductive axis, DNA methylation of *GnRH1* and time to reproduction. During normal development we saw no change in methylation between 14- and 28-day-old animals, however, levels of *GnRH1* mRNA remain relatively constant in the first several weeks after fertilization, increasing as the animal reaches sexual maturity [40]. The sharp increase in methylation seen in 6-week old animals may represent the transition between developmental and behavior-specific regulation. We hypothesize that as animals approach maturity, differential methylation may poise differential transcription of *GnRH1* specific to reproductive status. This is supported by examination of the specific CpG sites in the promoter that are differentially methylated during maturation. Interestingly, methylation at the 3 CpG sites that correspond to the putative binding site for *egr-1* (CpG -1313, -1310, and -1307) show no change during development. Expression of *egr-1* increases transiently when an adult animal ascends to dominant social status [27] and this specific location is differentially methylated during adult male social transitions (Lenkov et al, in review). It is important to note that our data represents average *GnRH1* methylation in the entire head of studies individuals. As there are less than 500 GnRH1 neurons in a single individual, our data could represent *GnRH1* methylation in some cells that *do not* express *GnRH1*. Therefore, direct comparison to studies of isolated GnRH1 containing cells can generalize that this loci-specific DNA methylation event corresponds to a global event that occurs throughout the tissue, thus requiring further validation. Furthermore, while we did not fully characterize a role for DNA methylation within the GnRH1 promoter, we speculate that the distribution of methylation around the transcription start site (TSS) may be indicative of its regulation of GnRH1 transcription. Hypermethylation of gene-body CpGs (+135, +199, and +207) isolated within the coding sequence of GnRH1(Fig 2B) are known to be associated with increased transcriptional activity [41, 42] as opposed to hypermethylation in distal areas relative to the TSS [43]. Future experiments measuring transcription at these time points as well as methylation reporter assays [44] will provide additional evidence in support of a role in transcription.

To ascertain the possible role of epigenetic mechanisms to adverse developmental conditions, we examined the effect of gestational crowding on differential methylation of *GnRH1*. Animals collected immediately after mouth brooding from crowded females had significantly lower levels of methylation in the promoter (-1410, -1146, -1128, and -1110 respectively). This was accompanied with increased transcription of *GnRH1* from crowded gestation, suggesting DNA hypomethylation occurring upstream of the TSS methylation may be regulating the transcription of GnRH1. Furthermore, in our crowded conditions, methylation and transcription may not be occurring within the exact same tissues and cell populations. Additional studies would be required to assay these molecular changes within single cells or cell types.

Even though these animals were carried by females during their development, the crowded conditions may have resulted in a change in behavior or physiology in the females which was transmitted to the brooding fry in her mouth and/or possibly to the oocytes before fertilization. There are a number of studies that have identified a correlation between prenatal maternal conditions and differential DNA methylation in their progeny. For example, in humans, children of mothers who were depressed during pregnancy have increased GR methylation [15]. Male offspring of mice exposed to gestational stress have decreased DNA methylation of the CRH gene promoter and increased methylation of the GR exon 1_7_ promoter region in hypothalamic tissue [45]. Social crowding also causes profound effects on tank environments which may work in concert with social behaviors to alter methylation. In this regard, cortisol accumulation and reduced quality of water may be direct consequences of crowding that in turn could affect methylation patterns [46].

There can be several reasons for this directional change in animals exposed either directly or indirectly to crowded conditions. Social crowding is likely to be stressful, and activation of the stress axis has an inhibitory effect on *GnRH1* expression. 42-day-old animals raised in crowded conditions were much smaller than their non-stress counterparts (Fig 2C), suggesting crowding further interferes with a number of developmental pathways. We suggest that these developmental pathways may be similar to those seen in male adult social status through somatostatin regulation [47]. Additionally, other reports have shown that genomic methylation can drive sizing continuums during development in a natural population, suggesting that genome wide levels of methylation must also be considered during early development [48].

Although DNA methylation was once thought to be involved only in the regulation of early embryonic development, recent studies of nutritional [49, 50], chemical [51], and other environmental factors [12, 45] which influence postnatal development have shown that epigenetic regulation of gene expression can be an important component in experience dependent change. Postnatal maternal separation in rodents is associated with decreased DNA methylation within the AVP promoter [52], while experience of low levels of licking and grooming in infancy is associated with increased methylation in the GR promoter region [14].

In many mammals and invertebrates the regulation of DNA methylation through diverse social cues is important for adapting to a novel environment. For example, regulating caste in wasps, bees and ants [53–55], and recovering from injury in mammals [56].

Our data expands on the repertoire of social interventions capable of regulating DNA methylation. Furthermore, we show a relationship between gestational crowding and decreased methylation of the *GnRH* 1 promoter and coding region. A survey of *GnRH1* methylation at several developmental time points reveals an increase in methylation between weeks 4 and 6 post-fertilization which is consistent with previous studies that have shown a connection between early-life events and epigenetic changes, suggesting that mechanism could be important for gene by environment interactions.

## Supporting Information

S1 Fig Comparing GnRH 1 methylation across promoter and coding regions in 42 day old animals between tissue from heads vs. bodies (t-test, p>0.05, n = 9–10 per group). Significance determined with an unpaired student’s T-test.
(TIF)
